# Nutritional and Metabolic Health Profiling in a Large Clinic-Based Sample of Mexican Adults: A Cross-Sectional Study

**DOI:** 10.3390/nu18111827

**Published:** 2026-06-05

**Authors:** Marco Antonio Luna-Ruiz-Esparza, Abraham García-Gil, Efren Encinas-Torres, Humberto Gómez-Campaña, Arely Sarahi Ramos-González, Diana Yadira Calva-Espinoza, Gerardo Benitez-Iturrios, Luis Fernando Hernández-Lezama, Abraham Campos-Romero, Jonathan Alcántar-Fernández

**Affiliations:** 1 Innovation and Research Department, Salud Digna, Culiacan 80184, Mexico; marco.luna@salud-digna.org (M.A.L.-R.-E.); abraham.gil@salud-digna.org (A.G.-G.); abraham.campos@salud-digna.org (A.C.-R.); 2 Salud Digna, Culiacan 80184, Mexico; eite59@yahoo.com.mx; 3 Encauza, Culiacan 80200, Mexico; 4 Medical Direction, Salud Digna, Culiacan 80184, Mexico; investigacion@salud-digna.org; 5 Nutrition Department, Salud Digna, Culiacan 80184, Mexico; arely.ramos@salud-digna.org; 6 Laboratory Department, Salud Digna, Culiacan 80184, Mexico; diana.calva@salud-digna.org (D.Y.C.-E.); gerardo.benitez@salud-digna.org (G.B.-I.); 7 Faculty of Law, National Autonomous University of Mexico, Mexico City 04510, Mexico; luisfhdez@ictinternacional.com

**Keywords:** nutrition, metabolic syndrome, prevalence, obesity

## Abstract

**Background/Objectives:** Obesity is a chronic, multifactorial condition characterized by excessive adipose tissue that adversely affects health and continues to rise worldwide. It is strongly associated with cardiometabolic abnormalities that increase the risk of adverse outcomes, including type 2 diabetes and coronary heart disease. **Methods:** We conducted a multicenter, clinic-based cross-sectional analysis of electronic health records from 200,022 adults aged ≥20 years, who accessed nutritional and clinical laboratory services at Salud Digna between 1 January 2022 and 31 December 2025. Nutritional status was classified as normal weight or overweight/obesity using body mass index criteria. Metabolic health was assessed using five components of the National Cholesterol Education Program Adult Treatment Panel III criteria. Individuals were defined as metabolically unhealthy if they met three or more metabolic syndrome criteria. **Results:** Among participants, 78.17% of males and 79.73% of females were classified as overweight or obese. Metabolic unhealthiness was observed in 50.74% of males and 55.42% of females. The prevalences of metabolically healthy normal weight, metabolically healthy overweight/obesity, metabolically unhealthy normal weight, and metabolically unhealthy overweight/obesity were 18.55%, 31.09%, 3.90%, and 44.20%, respectively. **Conclusions:** These findings highlight a high burden of overweight/obesity and metabolic abnormalities in a large clinic-based sample of Mexican adults. While not nationally representative, this study provides important insights into the distribution of nutritional and metabolic health profiles in individuals accessing healthcare services, supporting the need for targeted prevention, early detection, and management strategies in clinical settings.

## 1. Introduction

Obesity is a chronic, multifactorial disease defined by excessive adiposity that impairs physiological function and elevates the risk for numerous health complications [1]. Globally, the prevalence of overweight and obesity has risen steadily, driven by socio-economic development, rapid industrialization, increasingly sedentary lifestyles, reduced opportunities for physical activity, and the widespread availability of processed, energy-dense foods [2].

Excess body weight contributes to an estimated 1.6 million premature deaths annually from non-communicable diseases such as diabetes, cancer, cardiovascular disease, and stroke. Projections indicate that the global number of adults living with obesity will increase by more than 115% between 2010 and 2030—rising from 524 million to 1.13 billion—while childhood obesity among individuals aged 5–19 years is expected to grow by 60%, reaching 250 million by 2030 [3]. The economic implications are substantial as well: in 2019, overweight and obesity were estimated to account for 2.19% of global GDP [4].

In Mexico, the situation is particularly worrisome. Current estimates indicate that 36.1% of men and 41.6% of women live with obesity, and an additional 40.1% of men and 34.6% of women are classified as overweight [3]. These figures underscore the urgent need for effective public health interventions aimed at preventing and managing obesity and its associated metabolic consequences.

Individuals with obesity frequently exhibit a chronic condition of generalized biological stress, encompassing both systemic and adipose tissue inflammation. This condition is characterized by elevated levels of circulating inflammatory proteins and leukocytes, increased macrophage infiltration into subcutaneous adipose tissue, a higher proportion of proinflammatory CD4^+^ T cells, and upregulated expression of cytokine and chemokine genes [5,6,7]. Consequently, obesity is strongly associated with a wide spectrum of cardiometabolic abnormalities—including insulin resistance, β-cell dysfunction, non-alcoholic fatty liver disease, dyslipidemia, and hypertension—that collectively heighten the risk for major clinical outcomes such as type 2 diabetes and coronary heart disease [8].

Epidemiological studies play a critical role in evaluating patterns of nutritional status, given the substantial health implications associated with its variability. Considerable heterogeneity in nutritional status—from normal weight to obesity—can be observed across populations, and this variation becomes even more complex when metabolic health is taken into account. Comprehensive population profiling of nutritional and metabolic status is therefore essential for designing targeted intervention strategies aimed at mitigating the impact of obesity.

Utilizing data from Salud Digna—the largest clinical laboratory network in Mexico—we aimed to characterize the distribution of nutritional and metabolic health phenotypes in a large outpatient population and to examine variations by geography, sex, and age. While population-based surveys in Mexico, such as ENSANUT, provide nationally representative estimates of obesity and metabolic risk factors, they are primarily designed for population-level epidemiological surveillance and offer limited insight into the combined distribution of nutritional and metabolic phenotypes within clinical populations. In contrast, clinic-based surveillance reflects patterns among individuals actively accessing healthcare services and can provide complementary information relevant to clinical practice and health system planning.

An important knowledge gap therefore remains regarding how combined nutritional–metabolic phenotypes are distributed across body mass index categories in real-world healthcare settings, and how these patterns vary across demographic and geographic subgroups. Addressing this gap is critical to better understanding the heterogeneity of metabolic risk beyond conventional obesity measures and to informing targeted prevention and management strategies.

In this context, our clinic-based dataset provides distinct and complementary value. It enables the classification of individuals into detailed metabolic phenotypes using standardized biochemical and clinical measurements, allows for granular stratification by age, sex, and geographic region due to its large sample size, and captures individuals engaged in healthcare utilization. Together, these features offer novel insights into the burden and distribution of metabolic risk in a real-world clinical setting, complementing existing population-based evidence without aiming to provide nationally representative estimates.

## 2. Materials and Methods

### 2.1. Study Design and Participants

This multicenter, clinic-based cross-sectional study analyzed electronic health records from adults aged ≥20 years who accessed nutritional and clinical laboratory services at Salud Digna between 1 January 2022, and 31 December 2025.

### 2.2. Data Collection and Survey Instrument

Demographic and clinical information was obtained using a standardized questionnaire administered at the point of care across all Salud Digna facilities. This instrument collects information on age, sex, and basic clinical characteristics. The instrument is routinely used within the institution under standardized protocols to ensure consistency of data collection across sites.

Information on self-reported conditions, such as diabetes and hypertension, was collected through the same standardized survey instrument. These self-reported data were not independently verified against clinical diagnoses or medical records, and therefore were not used for metabolic classification, which was based exclusively on measured clinical and biochemical parameters.

### 2.3. Data Cleaning and Quality Control

A total of 666,480 records were initially screened.

Data cleaning procedures were applied sequentially to ensure data quality and analytical validity. Records with incomplete key anthropometric or biochemical measurements required for phenotype classification were first excluded (*n* = 447,715; 67.18%), leaving 218,765 records. Subsequently, records with data entry errors or implausible values were removed (*n* = 9348; 1.40%), resulting in 209,417 records.

Additional exclusions included individuals with underweight status (BMI < 18.5 kg/m^2^) and/or hypoglycemia (fasting glucose < 70 mg/dL) (*n* = 2749; 0.41%), leaving 206,668 records. Finally, records with values outside predefined physiological plausibility thresholds were excluded (*n* = 6646; 1.00%).

To minimize duplication, only the last available record per individual during the study period was retained for analysis. After all exclusions, the final analytical sample consisted of 200,022 unique individuals.

A complete-case approach was used, whereby only records with complete information for all required variables were included in the analysis.

### 2.4. Anthropometric and Clinical Measurements

All measurements were obtained by trained healthcare personnel following standardized protocols.

Body weight was measured using calibrated digital scales (InBody, Seoul, Republic of Korea), and height was measured using stadiometers, with participants wearing light clothing and no shoes. Body mass index (BMI) was calculated as weight in kilograms divided by height in meters squared (kg/m^2^).

Waist circumference was measured using a non-elastic measuring tape at the midpoint between the lower margin of the last rib and the iliac crest.

Blood pressure was measured using automated sphygmomanometers after a period of rest, following standard clinical procedures.

Blood samples were collected after an overnight fast of at least 8 h. Fasting glucose, triglycerides, and HDL cholesterol were measured using standardized laboratory methods. All laboratory analyses were conducted following internal quality control procedures and standardized protocols across participating laboratories.

### 2.5. Definition of Nutritional Status and Metabolic Health

Nutritional status was defined according to BMI categories as follows: normal weight (18.5–24.9 kg/m^2^), overweight (25.0–29.9 kg/m^2^), and obesity (≥30 kg/m^2^).

Metabolic health was assessed using the five components of the National Cholesterol Education Program Adult Treatment Panel III (NCEP ATP III) criteria for metabolic syndrome [5], detailed criteria is present in Table 1.

Only measured values were used for classification; information on prior diagnoses or medication use was not available and was therefore not incorporated into the definition.

The selected waist circumference was defined using cut-offs recommended for the Mexican population by national health authorities [6,7].

### 2.6. Nutritional–Metabolic Phenotypes

Participants were categorized into four combined phenotypes: metabolically healthy normal weight (MHNW), metabolically unhealthy normal weight (MUNW), metabolically healthy overweight/obesity (MHOO), and metabolically unhealthy overweight/obesity (MUOO) [8,9].

### 2.7. Statistical Analysis

All analyses were conducted using R statistical software (version 4.5.2) [10]. The analyses were primarily descriptive and exploratory in nature, aiming to characterize the distribution of nutritional and metabolic phenotypes within a large clinic-based population rather than to test predefined hypotheses.

The primary analyses estimated the clinic-based prevalence (prevalence within the clinic-attending population) of nutritional and metabolic health phenotypes using the epitools package (v0.5-10.1). To account for differences in age structure, prevalence estimates for Mexican states and by sex were age-standardized using the direct standardization method, with the World Health Organization (WHO) standard population as the reference [11]. Age groups were categorized as 20–24, 25–29, 30–34, 35–39, 40–44, 45–49, 50–54, 55–59, 60–64, 65–69, 70–74, 75–79, 80+ and standardized prevalence was calculated as the weighted sum of age-specific proportions using WHO standard population weights.

Exact 95% confidence intervals (CIs) for crude prevalence estimates were calculated using the Clopper–Pearson method. For age-standardized prevalence estimates, 95% CIs were derived using the normal approximation, given the large sample size.

Exploratory analyses included stratification by sex, age, and combined nutritional–metabolic phenotypes. Associations between categorical variables were examined using chi-square tests for independence (chisq.test() function in base R). Given the large sample size, *p*-values were interpreted cautiously, and greater emphasis was placed on effect size estimation. Effect size was quantified using Cramér’s V, with values interpreted as small (≤0.1), moderate (0.1–0.3), or strong (>0.3).

Due to the exploratory nature of the analyses and the large number of comparisons performed, no formal correction for multiple testing was applied. Instead, results were interpreted in conjunction with effect sizes and overall patterns, rather than relying solely on statistical significance.

Standardized rate ratios (SRRs) were calculated to compare prevalence between sexes. All statistical tests were two-sided, and a *p*-value <0.05 was considered statistically significant for descriptive purposes.

## 3. Results

Between 1 January 2022 and 31 December 2025, a total of 200,022 individuals aged 20 years or older accessed the nutritional and clinical laboratory services of Salud Digna and were included in the analysis.

Participants were drawn from 27 Mexican states, reflecting the geographic distribution of available clinical data within the study period. States not included had insufficient sample sizes to generate stable and reliable estimates of prevalence and were therefore excluded from state-level analyses. This should be considered when interpreting regional patterns.

The mean age of the study population was 48.3 years (SD ± 16.2), with 134,807 females (67.40%) and 65,215 males (32.60%). Among all participants, 23,638 individuals (11.82%) reported a previous diagnosis of diabetes, while 42,599 (21.30%) reported a prior diagnosis of high blood pressure.

The mean values for key clinical indicators were as follows: body mass index (BMI) 29.4 kg/m^2^, systolic blood pressure 123.2 mmHg, diastolic blood pressure 75.4 mmHg, fasting glucose 107.6 mg/dL, triglycerides 152.1 mg/dL, and HDL cholesterol 47.6 mg/dL. Table 2 summarizes the demographic and clinical characteristics of the study population.

### 3.1. Nutritional Status and Metabolic Health

**Figure 1:**
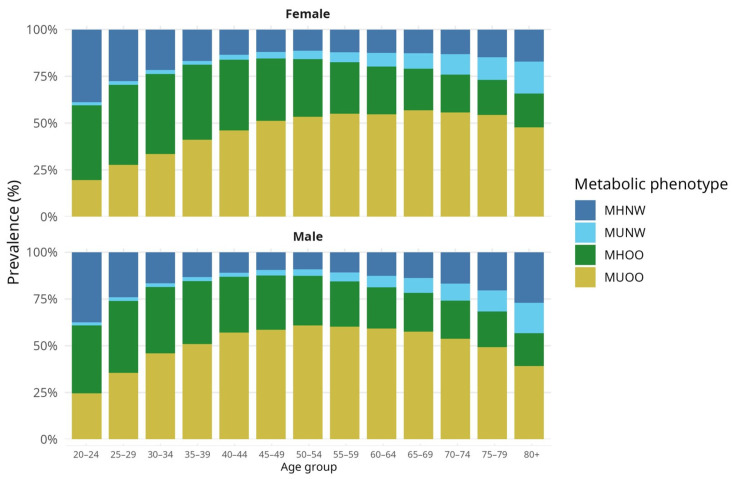
Age–sex distribution of individuals with the different nutritional and metabolic phenotypes. Abbreviations: MHNW: Metabolically healthy normal weight; MUNW: Metabolically unhealthy normal weight; MHOO: Metabolically healthy overweight/obese; MUOO: Metabolically unhealthy overweight/obese.

In this outpatient population, 78.17% of males and 79.73% of females were classified as overweight or obese, while 50.74% of males and 55.42% of females were metabolically unhealthy. A detailed breakdown of nutritional and metabolic phenotypes across sex and age groups is presented in Figure 1 and Table 3.

#### 3.1.1. Metabolically Healthy Normal Weight

**Figure 2:**
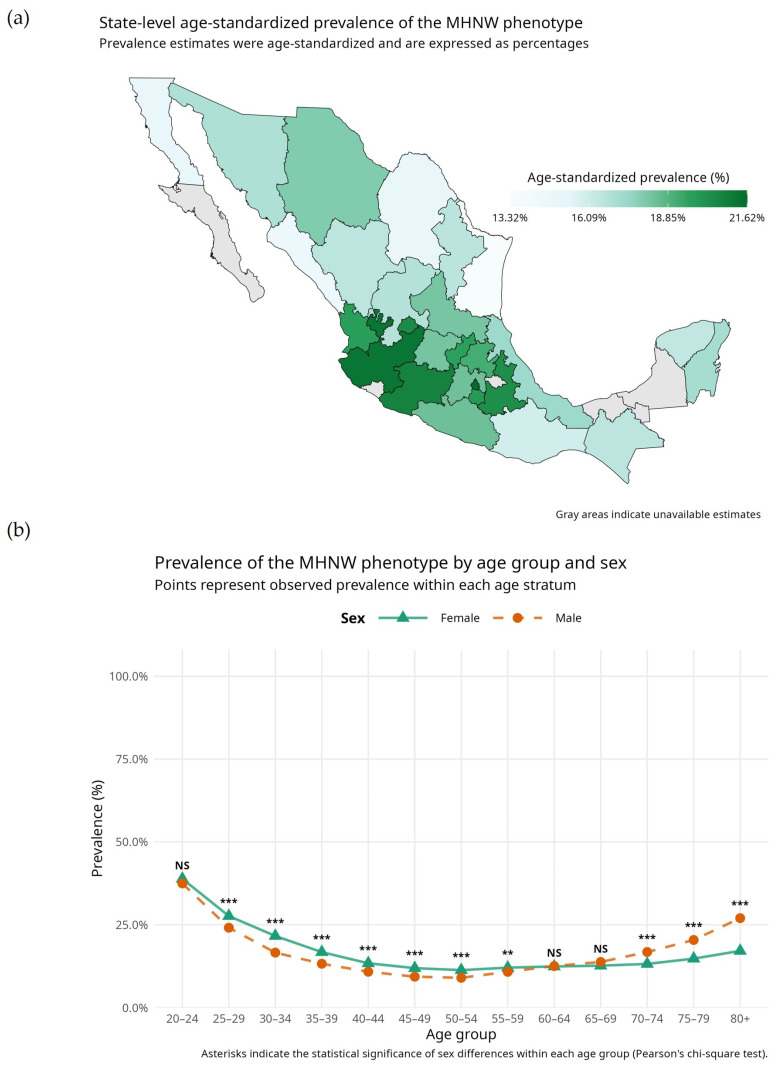
National (**a**) and age–sex (**b**) distribution of individuals with a metabolic healthy normal weight phenotype. Grey areas represent states with unavailable or insufficient data. Sex differences were evaluated using Pearson’s chi-square test without continuity correction. Significance codes: *** *p* < 0.001; ** *p* < 0.01, NS non-significant. Abbreviations: MHNW: Metabolically healthy normal weight.

The age-adjusted clinic-based prevalence (prevalence within the clinic-attending population) of the MHNW phenotype was 18.55% (95% CI: 18.37–18.74), with notable geographic variation, ranging from 13.32% in Tamaulipas to 21.62% (13.32%, 95% CI: 11.42–15.22) in Mexico City (95% CI: 21.13–22.11) (Figure 2a, Table S1).

Sex differences were statistically significant in several age groups, with higher prevalence in females between 25 and 59 years and in males ≥70 years (all *p* < 0.01). However, these differences were small in magnitude (Cramér’s V ≤ 0.1), suggesting limited clinical relevance despite statistical significance (Figure 2b, Table S2).

Across age groups, prevalence followed a consistent pattern, peaking in early adulthood (30–34 years) and declining markedly by midlife (50–54 years) in both sexes. When comparing sexes, females had a slightly higher standardized rate than males (SRR = 1.11, 95% CI: 1.09–1.13; *p* < 0.001), although the magnitude of this difference was modest.

#### 3.1.2. Metabolically Healthy Overweight/Obese

**Figure 3:**
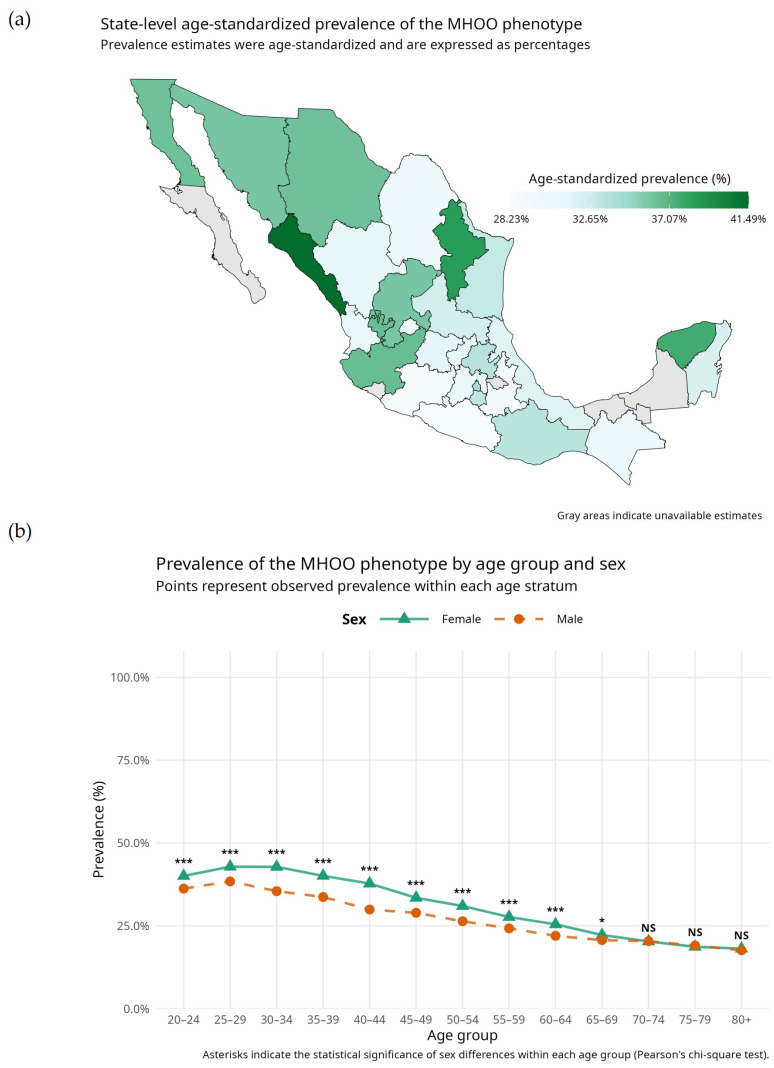
National (**a**) and age–sex (**b**) distribution of individuals with a metabolic healthy overweight/obese phenotype. Grey areas represent states with unavailable or insufficient data. Sex differences were evaluated using Pearson’s chi-square test without continuity correction. Significance codes: *** *p* < 0.001; * *p* < 0.05, NS non-significant. Abbreviations: MHOO: Metabolically healthy overweight/obese.

The age-adjusted clinic-based prevalence of the MHOO phenotype was 31.09% (95% CI: 30.89–31.29), with substantial geographic variation, ranging from 23.83% (95% CI: 22.13–25.59) in Guerrero to 39.01% in Sinaloa (95% CI: 37.98–40.04) (Figure 3a, Table S1).

Females had a higher prevalence than males across most age groups (20–64 years and 65–69 years; all *p* < 0.05). However, effect size estimates indicated that these differences were small (Cramér’s V < 0.1), suggesting limited clinical relevance despite statistical significance (Figure 3b, Table S2).

Across the life course, prevalence peaked in early adulthood (25–29 years) and declined steadily with advancing age, reaching its lowest levels in individuals ≥ 80 years. Although females had a higher overall standardized rate than males (SRR = 1.15, 95% CI: 1.14–1.17; *p* < 0.001), the magnitude of this difference was modest.

#### 3.1.3. Metabolically Unhealthy Normal Weight

**Figure 4:**
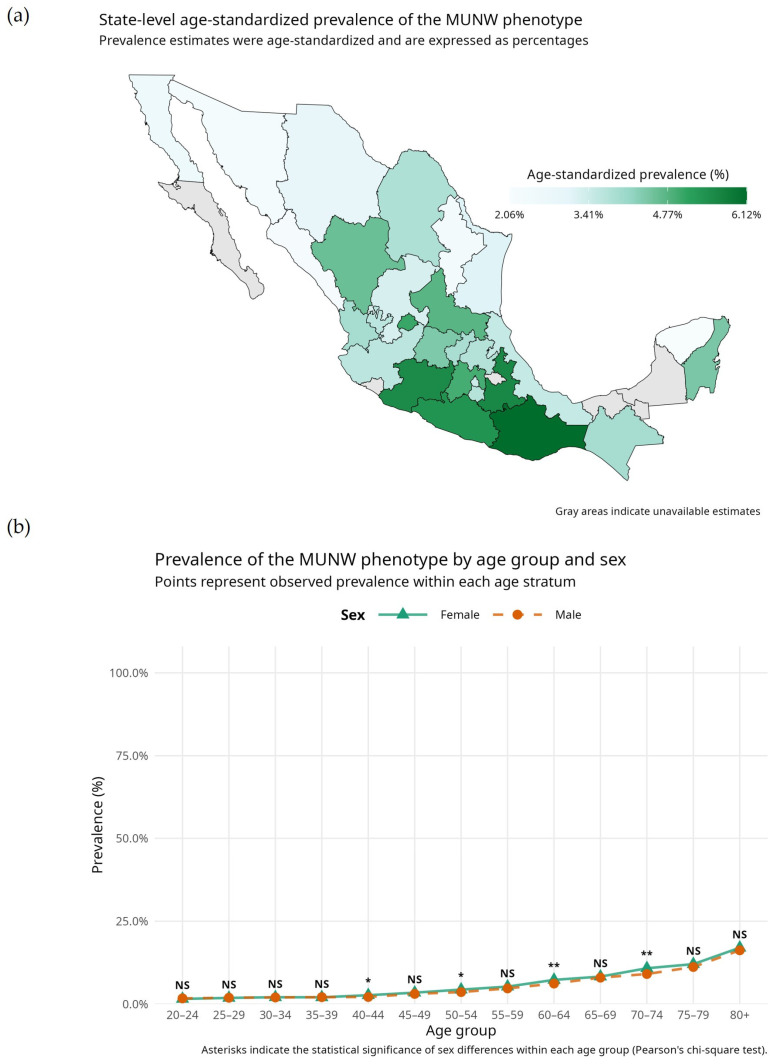
National (**a**) and age–sex (**b**) distribution of individuals with a metabolic unhealthy normal weight phenotype. Grey areas represent states with unavailable or insufficient data. Sex differences were evaluated using Pearson’s chi-square test without continuity correction. Significance codes: * *p* < 0.05, ** *p* < 0.01 NS non-significant. Abbreviations: MUNW: Metabolically unhealthy normal weight.

The age-adjusted clinic-based prevalence of the MUNW phenotype was 3.90% (95% CI: 3.82–3.98), showing geographic variability, with the highest prevalence in Oaxaca (6.12%, 95% CI: 4.15–8.09) and the lowest in Yucatan (2.06%, 95% CI: 1.28–2.84)) (Figure 4a, Table S1).

Sex differences were observed in selected age groups, with higher prevalence in females in mid- and older adulthood (40–74 years; *p* < 0.05). However, these differences were small in magnitude (Cramér’s V < 0.1), indicating limited clinical relevance (Figure 4b, Table S2).

Across age groups, prevalence increased steadily with age, reaching its highest levels in individuals ≥ 80 years, while remaining low in young adults (20–24 years). Although females had a slightly higher overall standardized rate than males (SRR = 1.09, 95% CI: 1.04–1.14; *p* < 0.001), the magnitude of this difference was modest.

#### 3.1.4. Metabolically Unhealthy Overweight Obese

**Figure 5:**
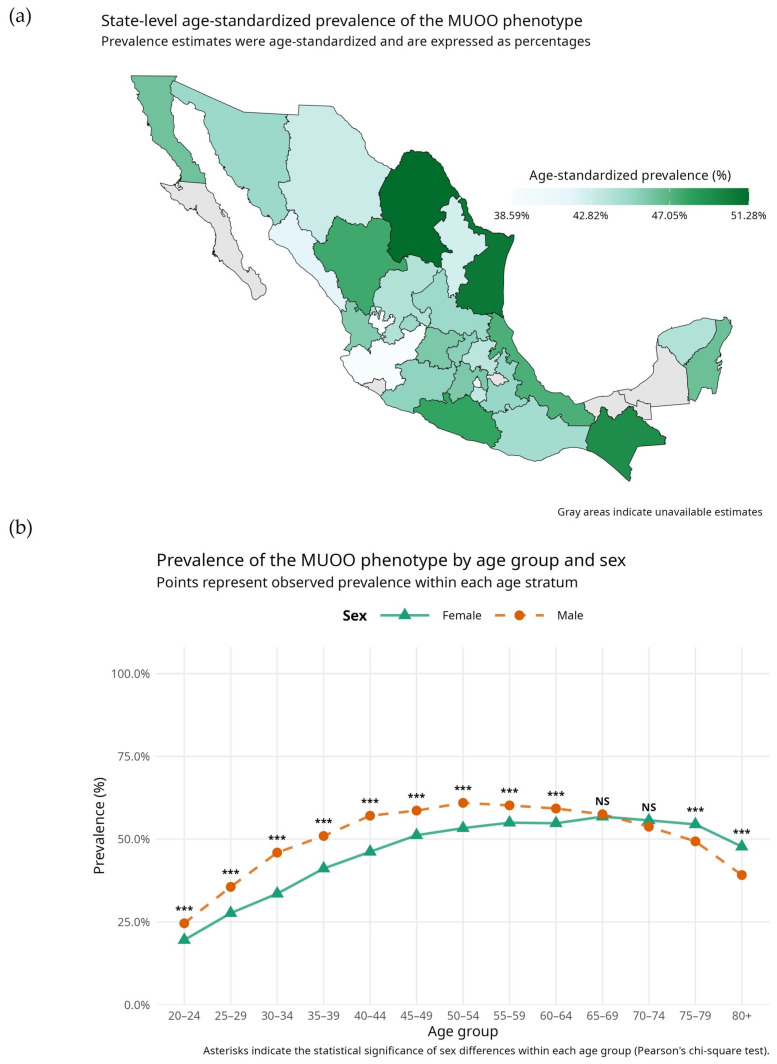
National (**a**) and age–sex (**b**) distribution of individuals with a metabolic unhealthy overweight/obese phenotype. Grey areas represent states with unavailable or insufficient data. Sex differences were evaluated using Pearson’s chi-square test without continuity correction. Significance codes: *** *p* < 0.001, NS non-significant. Abbreviations: MUOO: Metabolically unhealthy overweight/obese.

The age-adjusted clinic-based prevalence of the MUOO phenotype was 44.20% (95% CI: 43.98–44.42), making it the most prevalent phenotype in the study population. Geographic variation was observed, ranging from 38.59% (95% CI: 37.77–39.40) in Jalisco to 51.28% (95% CI: 48.60–53.95) in Coahuila (Figure 5a, Table S1).

Sex differences were statistically significant across age groups, with higher prevalence in males during early and middle adulthood (20–64 years) and in females at older ages (≥75 years) (all *p* < 0.001). However, consistent with other phenotypes, these differences were small in magnitude (Cramér’s V ≤ 0.1), indicating limited clinical relevance (Figure 5b, Table S2).

Across the life course, prevalence increased sharply from young adulthood, reaching a peak in mid-to-late adulthood (earlier in males and later in females), and declined slightly thereafter. Overall, males had a higher standardized rate than females (SRR = 0.86, 95% CI: 0.85–0.87; *p* < 0.001), although the magnitude of this difference was modest.

To further characterize the distribution of metabolic abnormalities across phenotypes, we examined the contribution of individual components (Figure S1). Overall, the distribution of glucose and lipid abnormalities was similar between the MUNW and MUOO phenotypes. However, abdominal obesity was markedly more prevalent in the MUOO group compared with MUNW. In contrast, within the MUNW group, the distribution of metabolic components was relatively balanced, with no single abnormality disproportionately dominant.

These findings highlight differences in the composition of metabolically unhealthy phenotypes across BMI categories, with central adiposity showing the greatest distinction between normal weight and overweight/obesity groups.

Overall, the distribution of metabolic phenotypes revealed distinct but complementary patterns across the life course. Metabolically healthy phenotypes (MHNW and MHOO) were more prevalent in younger adults and declined with age, whereas metabolically unhealthy phenotypes—particularly MUOO—became increasingly dominant in midlife, representing the most prevalent clinical profile. In contrast, MUNW, although less frequent, showed a progressive increase with advancing age, suggesting a shifting metabolic risk among individuals with normal weight. While several age- and sex-related differences were statistically significant, their effect sizes were consistently small, indicating limited clinical impact at the population level. Together, these findings highlight a dynamic transition in metabolic health across age groups, with important implications for the timing and targeting of preventive strategies.

## 4. Discussion

In this large clinic-based study of Mexican adults, we identified distinct patterns in the distribution of nutritional and metabolic phenotypes across the life course. Metabolically healthy phenotypes were more prevalent in younger individuals, whereas metabolically unhealthy phenotypes—particularly metabolically unhealthy overweight/obesity (MUOO)—were most common in midlife. In contrast, metabolically unhealthy normal weight (MUNW), although less frequent, increased with advancing age. While several age- and sex-related differences were statistically significant, their effect sizes were small, indicating limited clinical relevance at the individual level. Importantly, these findings reflect patterns observed within a self-selected outpatient population and provide insight into the burden and heterogeneity of metabolic risk in real-world clinical settings.

Our clinic-based estimates of overweight, obesity, and metabolic syndrome were higher than those reported in national population-based surveys (e.g., ENSANUT) [12,13,14,15]. This difference is likely attributable, at least in part, to selection bias, as the dataset reflects a self-selected population potentially enriched for individuals with existing health concerns or greater health-seeking behavior. Therefore, direct comparison with nationally representative data should be interpreted with caution. Nonetheless, the high burden observed in this large clinical population highlights the substantial demand for metabolic health services.

Geographic variation in the distribution of nutritional and metabolic phenotypes was evident, with a higher prevalence of metabolically unhealthy phenotypes in northeastern and southern states. These patterns may be associated with differences in environmental exposures, socioeconomic conditions, and access to healthcare services, which have previously been linked to metabolic risk in Mexico [16,17,18]. However, these interpretations should be considered exploratory, as the present study was not designed to directly assess these factors. Therefore, these findings do not establish causal relationships and instead highlight potential contextual influences that warrant further investigation in future studies.

The higher prevalence of the MUNW phenotype in southern states, which are also characterized by greater socioeconomic disadvantage [19], warrants particular attention. This pattern may reflect a complex interaction of nutritional, environmental, and healthcare access factors. Additionally, cultural perceptions of body weight could influence risk awareness and healthcare-seeking behavior, although these hypotheses cannot be directly evaluated in the present study.

Importantly, the disaggregation of metabolic components revealed that the overall profile of metabolic abnormalities in the MUNW group was broadly similar to that observed in the MUOO phenotype. In both groups, glucose and lipid alterations were comparably distributed; however, abdominal obesity was markedly more prevalent in the MUOO group. This suggests that, while metabolic dysfunction is present regardless of overall body weight, central adiposity may represent a key distinguishing factor between these phenotypes.

These findings reinforce the limitations of BMI as a sole indicator of metabolic health and highlight that individuals with normal weight but adverse metabolic profiles may share a risk pattern comparable to those with overt obesity. Consequently, reliance on BMI alone may lead to underrecognition of at-risk individuals, underscoring the importance of incorporating measures of abdominal adiposity and metabolic screening across all weight categories.

The relatively high prevalence of the MHOO phenotype, particularly among younger individuals, suggests that a substantial proportion of individuals with excess weight may not yet exhibit overt metabolic abnormalities. Prior longitudinal studies have shown that transitions from metabolically healthy to unhealthy states can occur over time [8]. However, given the cross-sectional design of the present study, such transitions cannot be directly assessed. Therefore, the observed age-related patterns are consistent with the hypothesis that some individuals with MHOO may progress toward MUOO, but this interpretation should be considered speculative and requires confirmation in longitudinal studies.

Although sex-based differences in overweight and obesity prevalence were modest, the MUOO phenotype was more frequent among males in younger age groups. This finding differs from global patterns, where obesity is often more prevalent in women [20]. One possible explanation is that men may be underrepresented in preventive healthcare settings and more likely to seek care at later stages of disease [21]; however, this interpretation should be considered cautiously, as healthcare-seeking behavior was not directly assessed in this study.

Taken together, these findings highlight the importance of early detection and phenotype-based risk stratification across the course of life. While the present study identifies important patterns in a large clinical population, further longitudinal and population-based research is needed to better understand the determinants and progression of metabolic phenotypes, and to inform targeted and context-appropriate public health interventions.

Effective obesity management requires individualized, phenotype-oriented approaches. In the present study, the high prevalence of metabolically unhealthy phenotypes—particularly MUOO in midlife—highlights the need for early, targeted interventions in individuals with both excess adiposity and metabolic dysfunction. Conversely, the presence of metabolically unhealthy individuals with normal weight (MUNW), especially at older ages, underscores the limitations of relying solely on body mass index and supports the need for metabolic screening across all weight categories. In this context, nutrition therapy guided by trained professionals plays a critical role in risk stratification and management. Even modest weight loss (≥5%) has been shown to produce clinically meaningful improvements in cardiometabolic risk, with greater benefits observed at ≥10% weight loss [22].

Exercise has well-documented benefits, including improved insulin sensitivity, enhanced metabolic function, and a reduction in systemic inflammation [23]. Within the context of our findings, promoting physical activity from early adulthood may be particularly relevant to delaying or mitigating the transition toward more adverse metabolic profiles observed in midlife and older age.

This study has limitations that should be considered when interpreting the findings. First, the use of a self-selected outpatient population introduces the potential for selection bias, as individuals accessing clinical laboratory services may differ systematically from the general population in terms of health status and healthcare-seeking behavior. In addition, a large proportion of records (67.18%) were excluded due to incomplete key measurements required for phenotype classification, which may further introduce selection bias and affect the interpretation and generalizability of the findings. Consistent with this, the absence of sampling weights and the clinic-based design preclude national representativeness and limit generalizability.

Second, the dataset includes an overrepresentation of women and does not encompass all Mexican states, which may affect the observed distributions of metabolic phenotypes.

Additionally, the use of electronic health records may introduce data-related limitations, including the possibility of duplicate records, missing information, and variability in data completeness across sites. The lack of information on medication use is particularly relevant, as pharmacological treatment may influence metabolic parameters and phenotype classification. Furthermore, all measurements were obtained at a single time point, which limits the ability to assess temporal changes or transitions between metabolic states. Finally, due to the cross-sectional design, causal relationships cannot be established, and all findings should be interpreted as descriptive associations.

## 5. Conclusions

The high prevalence of overweight/obesity and metabolic abnormalities observed in this study highlights the substantial burden of cardiometabolic risk within a large clinic-based population of Mexican adults. These findings underscore the importance of identifying metabolic risk beyond body mass index alone, particularly given the heterogeneity of nutritional–metabolic phenotypes across age and sex.

Within the context of a clinic-based setting, our results provide relevant insights to inform institutional strategies for screening, early detection, and follow-up care. In particular, the identification of both metabolically unhealthy individuals with excess weight and those with normal weight supports the need for comprehensive metabolic evaluation across all patient groups. These findings may help guide the development and optimization of targeted prevention and management programs in similar healthcare environments.

## Figures and Tables

**Table 1:** Criteria used for metabolic syndrome classification.

Individuals meeting three or more of these criteria were classified as metabolically unhealthy.	
1. Elevated blood pressure	
- Elevated systolic blood pressure	≥130 mmHg
- Elevated diastolic blood pressure	≥85 mmHg
2. Elevated fasting glucose	≥100 mg/dL (5.6 mmol/L)
3. Elevated triglycerides	≥150 mg/dL (1.7 mmol/L)
4. Reduced HDL cholesterol:	
- Women	<50 mg/dL (1.3 mmol/L)
- Men	<40 mg/dL (1.0 mmol/L)
5. Central obesity (waist circumference in cm)	
- Women	>80 cm in women
- Men	>90 cm

**Table 2:** Baseline demographic and clinical characteristics of the study population, overall and by sex. Values are presented as *n* (%) unless otherwise indicated; continuous variables are presented as mean (SD).

Characteristic	Overall (*n* = 200,022)	Female (*n* = 134,807)	Male (*n* = 65,215)
Sex			
Female	134,807 (67.40%)	134,807 (100.00%)	0 (0.00%)
Male	65,215 (32.60%)	0 (0.00%)	65,215 (100.00%)
Age, years			
Mean (SD)	48.3 (16.2)	48.6 (16.1)	47.6 (16.4)
Age group			
20–24	16,123 (8.06%)	10,793 (8.01%)	5330 (8.17%)
25–29	17,201 (8.60%)	11,166 (8.28%)	6035 (9.25%)
30–34	17,101 (8.55%)	10,982 (8.15%)	6119 (9.38%)
35–39	15,055 (7.53%)	9439 (7.00%)	5616 (8.61%)
40–44	16,713 (8.36%)	10,865 (8.06%)	5848 (8.97%)
45–49	19,608 (9.80%)	13,534 (10.04%)	6074 (9.31%)
50–54	22,313 (11.16%)	15,783 (11.71%)	6530 (10.01%)
55–59	20,717 (10.36%)	14,512 (10.77%)	6205 (9.51%)
60–64	19,326 (9.66%)	13,340 (9.90%)	5986 (9.18%)
65–69	15,708 (7.85%)	10,809 (8.02%)	4899 (7.51%)
70–74	10,311 (5.15%)	7039 (5.22%)	3272 (5.02%)
75–79	5972 (2.99%)	4015 (2.98%)	1957 (3.00%)
80+	3874 (1.94%)	2530 (1.88%)	1344 (2.06%)
Diabetes			
No	176,384 (88.18%)	118,768 (88.10%)	57,616 (88.35%)
Yes	23,638 (11.82%)	16,039 (11.90%)	7599 (11.65%)
High blood pressure			
No	157,423 (78.70%)	105,533 (78.28%)	51,890 (79.57%)
Yes	42,599 (21.30%)	29,274 (21.72%)	13,325 (20.43%)
Body mass index, kg/m^2^			
Mean (SD)	29.4 (5.4)	29.5 (5.7)	29.1 (4.9)
Systolic blood pressure, mmHg			
Mean (SD)	123.2 (16.1)	121.4 (15.8)	126.8 (16.0)
Diastolic blood pressure, mmHg			
Mean (SD)	75.4 (10.1)	73.7 (9.6)	78.7 (10.3)
Fasting glucose, mg/dL			
Mean (SD)	107.6 (41.8)	105.9 (38.9)	111.1 (47.0)
Triglycerides, mg/dL			
Mean (SD)	152.1 (91.5)	145.0 (82.0)	166.8 (107.0)
HDL cholesterol, mg/dL			
Mean (SD)	47.6 (13.2)	50.2 (13.3)	42.2 (11.0)

**Table 3:** Prevalence of nutritional and metabolic phenotypes by age and sex.

			Prevalence of the Different Nutritional and Metabolic Phenotypes %							
			MHNW		MUNW		MHOO		MUOO	
Age (y)	Sex	Total	*n*	%	*n*	%	*n*	%	*n*	%
20–24	Female	10,793	4194	38.86	169	1.57	4319	40.02	2111	19.56
	Male	5330	1997	37.47	92	1.73	1930	36.21	1311	24.6
25–29	Female	11,166	3087	27.65	209	1.87	4781	42.82	3089	27.66
	Male	6035	1456	24.13	116	1.92	2316	38.38	2147	35.58
30–34	Female	10,982	2377	21.64	228	2.08	4696	42.76	3681	33.52
	Male	6119	1018	16.64	123	2.01	2169	35.45	2809	45.91
35–39	Female	9439	1584	16.78	194	2.06	3781	40.06	3880	41.11
	Male	5616	746	13.28	118	2.1	1892	33.69	2860	50.93
40–44	Female	10,865	1458	13.42	292	2.69	4100	37.74	5015	46.16
	Male	5848	636	10.88	126	2.15	1749	29.91	3337	57.06
45–49	Female	13,534	1618	11.96	467	3.45	4526	33.44	6923	51.15
	Male	6074	570	9.38	187	3.08	1757	28.93	3560	58.61
50–54	Female	15,783	1792	11.35	690	4.37	4888	30.97	8413	53.3
	Male	6530	591	9.05	237	3.63	1722	26.37	3980	60.95
55–59	Female	14,512	1757	12.11	765	5.27	4015	27.67	7975	54.95
	Male	6205	673	10.85	294	4.74	1504	24.24	3734	60.18
60–64	Female	13,340	1662	12.46	975	7.31	3396	25.46	7307	54.78
	Male	5986	753	12.58	371	6.2	1316	21.98	3546	59.24
65–69	Female	10,809	1373	12.7	896	8.29	2403	22.23	6137	56.78
	Male	4899	677	13.82	391	7.98	1014	20.7	2817	57.5
70–74	Female	7039	931	13.23	761	10.81	1429	20.3	3918	55.66
	Male	3272	550	16.81	297	9.08	667	20.39	1758	53.73
75–79	Female	4015	595	14.82	484	12.05	750	18.68	2186	54.45
	Male	1957	400	20.44	219	11.19	373	19.06	965	49.31
80+	Female	2530	434	17.15	430	17	459	18.14	1207	47.71
	Male	1344	363	27.01	218	16.22	237	17.63	526	39.14
Overall	Female	134,807	22,862	16.96	6560	4.87	43,543	32.3	61,842	45.87
	Male	65,215	10,430	15.99	2789	4.28	18,646	28.59	33,350	51.14

## Data Availability

The data supporting the findings of this study are available from the corresponding author upon reasonable request. Owing to legal and ethical restrictions under the Mexican Federal Law on Protection of Personal Data Held by Private Parties, access to the data is limited to ensure the privacy and confidentiality of the study participants. Requests will be evaluated according to institutional policies and applicable regulations.

## Supplementary Materials

The following supporting information can be downloaded at: https://www.mdpi.com/article/10.3390/nu18111827/s1, Figure S1: Proportion of metabolic syndrome components by phenotypes, Table S1: Crude and age-adjusted prevalence of nutritional and metabolic phenotypes by state of residence, Table S2: Prevalence of nutritional and metabolic phenotypes by age and sex, with sex-based comparisons within each age stratum, Table S3: Sex-specific age-standardized rates and standardized rate ratios by metabolic phenotype, Table S4: Prevalence of nutritional and metabolic phenotypes by age and sex, with sex-based comparisons within each age stratum.

## Abbreviations

The following abbreviations are used in this manuscript:

MHNW	Metabolically Healthy Normal Weight
MHOO	Metabolically Healthy Overweight/Obese
MUNW	Metabolically Unhealthy Normal Weight
MUOO	Metabolically Unhealthy Overweight/Obese
